# Elevated fibroblast growth factor 23 levels predict cardiovascular and cerebrovascular events in acute ischaemic stroke patients

**DOI:** 10.3389/fneur.2026.1777221

**Published:** 2026-03-20

**Authors:** Zhuo Chen, Haoran Chen, Lingyun Gu, Zhuowen Xu, Weizhang Li, Junyou Cui, Hua Zhang

**Affiliations:** 1 The Affiliated Jiangyin Clinical College of Xuzhou Medical University, Jiangyin, China; 2 Department of Cardiology, Jiangyin Hospital Affiliated to Nantong University, Jiangyin, China

**Keywords:** acute ischaemic stroke, biomarker, fibroblast growth factor 23, major adverse cardiovascularand cerebrovascular events, prognostic prediction

## Abstract

**Background:**

Recent studies on fibroblast growth factor 23 (FGF23) and acute ischaemic stroke (AIS) have been conducted, but some of the results are conflicting. Therefore, the objective of this study was to assess whether baseline FGF23 levels are associated with the occurrence of major adverse cardiovascular and cerebrovascular events (MACCEs) in AIS patients.

**Methods:**

In this study, 394 patients with AIS were enrolled. A total of 130 people who underwent health check-ups in our hospital composed the control group. The study endpoint was the first occurrence of MACCEs outside the hospital. Serum FGF23 levels were measured using a commercial human enzyme-linked immunosorbent assay kit.

**Results:**

Eighty five MACCEs were recorded during a median follow-up of 43 months. Serum FGF23 levels were found to be significantly greater in the AIS group than in the control group (525.14 ± 167.40 pg./mL vs. 338.62 ± 161.71 pg./mL, *p* < 0.05). Furthermore, the serum FGF23 level was greater in the MACCE group than in the non-MACCE group (646.09 ± 164.05 pg./mL vs. 491.87 ± 152.54 pg./mL, *p* < 0.05). According to the receiver operating characteristic curve analysis, the predictive value of the combination of the three indicators [the FGF23 level, National Institutes of Health Stroke Scale (NIHSS) score and modified Rankin scale (mRS) score] for the occurrence of MACCEs in AIS patients was greater than that of any of them alone (*p* < 0.05), while the difference between the three indicators was not statistically significant. The cumulative MACCE-free survival was found to be significantly greater in the group with low FGF23 levels than in the group with high FGF23 levels, as demonstrated by Kaplan–Meier analysis (*p* < 0.05). The multivariate Cox analysis revealed that the elevated baseline serum FGF23 levels (HR: 3.731, 95% CI: 2.157–6.452) were a significant predictor of MACCEs in AIS patients.

**Conclusion:**

Elevated baseline serum FGF23 level was considered a valid predictor of MACCEs in patients with AIS.

## 1 Introduction

Acute ischaemic stroke (AIS), a common clinical neurological disorder, is the second leading cause of disability and death worldwide (1). AIS patients are at high risk of major adverse cardiovascular and cerebrovascular events (MACCEs), resulting in a significant socioeconomic burden (2–4). A study revealed that the cumulative risk of recurrence of thrombotic arterial events, including the recurrent AIS, cardiovascular events and death, was greater than 11% within 5 years of the first occurrence of AIS (5). The risk of AIS recurrence is about 15% 10 years after AIS onset, and the risk of hospitalisation for cardiovascular events and AIS recurrence 15 years later is approximately 20% (6–8). Therefore, the early identification and aggressive treatment of AIS patients at high risk of developing MACCEs are clinically important.

Although multiple methods can be used to predict the prognosis of AIS, their predictive efficacy is limited. However, biomarkers may provide additional information to existing predictive methods to guide clinical practice (9). Several biomarkers, such as osteoprotegerin, C-reactive protein, galectin-3, and brain-derived neurotrophic factor, are associated with the prognosis of AIS and have good predictive value (10–13). In recent years, studies related to the role of fibroblast growth factor 23 (FGF23) in cerebrovascular disease have received increasing attention.

FGF23 is a phosphotropic hormone that is secreted mainly by osteoblasts and osteoclasts and acts in various organs via endocrine mechanisms (14). FGF23 in the blood can be transported into the brain through the blood–brain barrier or the blood-cerebrospinal fluid barrier (15). Previous studies have shown that FGF23 levels are associated with the severity and prognosis of a variety of diseases, including diabetes mellitus, chronic coronary heart disease, heart failure and chronic kidney disease (16–19).

Recent studies revealed that serum FGF23 levels were significantly elevated in patients with AIS and correlated with short-term prognosis (20). The North Manhattan study revealed that elevated FGF23 levels were a risk factor for stroke and cerebral haemorrhage events in the population and were associated with small vessel disease and cerebral infarction but not with chronic kidney disease (21, 22). A meta-analysis of seven high-quality prospective studies revealed a significant positive association between baseline FGF23 levels and stroke incidence and that elevated FGF23 levels were an independent predictor of stroke (23). Moreover, basic research has confirmed that dysregulation of FGF23 increases vascular stiffness, promotes inflammatory responses, vascular calcification and atherosclerosis—all of which are key mechanisms in the development and progression of stroke (24–27).

However, there are still inconsistencies in the findings of studies correlating FGF23 levels with the risk of developing AIS. The Multi-Ethnic Atherosclerosis Study (MESA), which included 6,547 members of the general population initially free of cardiovascular disease, revealed that FGF23 levels were not associated with the risk of developing stroke (28). The Reasons for Geographic and Racial Differences in Stroke (REGARDS) study, which included 615 stroke patients, revealed that elevated levels of FGF23 were associated with the risk of developing cardioembolic stroke, but not with the risk of developing other stroke subtypes, in community-dwelling adults (29). These inconsistent findings may stem from multiple sources of heterogeneity across studies, including baseline differences in study populations, variations in stroke subtype classification, variations in follow-up duration, and differences in outcome definitions. Additionally, the method used for FGF23 measurement can affect the comparability of study results. Notably, few studies have assessed the correlations between FGF23 levels and the occurrence of MACCEs in AIS patients. Therefore, the purpose of this study was to assess whether baseline FGF23 levels are associated with the risk of developing AIS and are useful for predicting the occurrence of MACCEs.

## 2 Materials and methods

### 2.1 Study population

This was a prospective study. Three hundred ninety-four patients who met the definition of AIS in the Chinese AIS Diagnostic and Treatment Guidelines for AIS were enrolled (30). All these patients received standardised treatment according to the guidelines between January 2020 and September 2020 at the Affiliated Jiangyin Clinical College of Xuzhou Medical University. A total of 130 patients in the general population who underwent physical examination during the same period composed the control group. The exclusion criteria were as follows: recurrent ischaemic stroke or cerebral haemorrhage with neurodegenerative diseases such as Parkinson’s disease, Alzheimer’s disease or vascular dementia; transient ischaemic attack; haematological diseases; autoimmune diseases; coinfections; end-stage renal disease; and inability to follow up.

This study was obtained approval from the Ethics Committee of Jiangyin People’s Hospital (Approval No. 2020008). Informed consent was obtained from the patients or their families (patients who were unable to communicate) prior to enrolment of all patients.

### 2.2 Clinical and laboratory assessments

After the patients were enrolled, a detailed neurological physical examination was performed and recorded after detailed questioning of their current and past medical history by experts with extensive clinical experience. The height and weight of the patients were recorded to calculate the body mass index (BMI). The National Institutes of Health Stroke Scale (NIHSS) score was used to assess the severity of AIS immediately after patient enrolment (31). The Oxfordshire Community Stroke Project criteria and the Trial of Org 10172 in Acute Stroke Treatment (TOAST) classification criteria were used to identify stroke syndromes and stroke aetiology, respectively, in the enrolled patients, respectively (32).

Elbow venous blood was drawn from fasting patients on the morning of the second day of hospitalisation. A portion of the blood sample was delivered to the central laboratory where total cholesterol, triglycerides, low-density lipoprotein cholesterol (LDL-C), high-density lipoprotein cholesterol (HDL-C), and creatinine levels were measured using the Roche e602 and c701 modules, respectively. The other part of the blood sample was left at room temperature for 1 h and then centrifuged at 3000 r/min for 15 min to obtain the upper serum layer, which was stored at −80 °C in a refrigerator. After following the instructions of the human enzyme-linked immunosorbent assay kit (Elabscience), which has a detection range of 15.63–1,000 pg./mL, a sensitivity of <9.38 pg./mL, and intra- and inter-assay coefficients of variation of <10 and <10%, respectively, the serum FGF23 concentration was measured at 450 nm using an enzyme marker.

### 2.3 Follow-up

Patients or their families were followed up monthly through clinic visits or by telephone. The median follow-up time in this study as of December 2023 was 43 months (interquartile range, 39–44 months). The endpoint event in this study was the first out-of-hospital MACCE, which included AIS, lacunar cerebral infarction, cerebral haemorrhage, non-fatal myocardial infarction, heart failure, and all-cause mortality. At the end of the follow-up, a modified Rankin scale (mRS) was used to assess the neurological recovery of the enrolled patients.

### 2.4 Statistical analysis

Statistical analysis was performed using the SPSS 25.0 statistical package. Quantitative variables were assessed for normality using the Kolmogorov–Smirnov test and were expressed as the means ± standard deviations and were compared using Student’s *t*-test. Categorical variables were expressed as absolute numbers (percentages) and were compared using the chi-square test. The receiver operating characteristic (ROC) curve was used to determine the optimal cut-off value for each variable to predict the occurrence of MACCEs in patients with AIS. Comparisons between different ROC curves were Z tested by MedCalc software. Kaplan–Meier survival curves were used for survival analysis and compared using the log-rank test. Univariate and multivariate Cox proportional hazards models were used to analyse the relationships between serum FGF23 levels and the occurrence of MACCEs, and hazard ratios (HR) and 95% confidence intervals (CI) were calculated. All variables that reached statistical significance in the univariate Cox analysis were included in the multivariate Cox analysis. A two-tailed *p* value < 0.05 was considered to indicate statistical significance.

## 3 Results

### 3.1 Baseline characteristics of the study participants

Comparisons of demographic and baseline clinical characteristics between the AIS and control groups are shown in Table 1. Serum FGF23 levels were significantly greater in the AIS group than in the control group (525.14 ± 167.40 pg./mL vs. 338.62 ± 161.71 pg./mL, *p* < 0.05). The prevalence of hypertension, diabetes mellitus and atrial fibrillation was significantly greater in the AIS group than in the control group (*p* < 0.05). There was also a statistical difference between the two groups in terms of BMI and blood LDL-C levels, whereas there was no statistical difference in terms of sex, age, blood total cholesterol, triglyceride, HDL-C or creatinine levels.

**Table 1:** The demographic and baseline clinical characteristics of the patients.

Variables	AIS group (*n* = 394)	Control group (*n* = 130)	*p*
Male, *n* (%)	236 (59.90%)	80 (61.54%)	0.740
Age (years)	67.32 ± 12.29	65.70 ± 11.42	0.186
BMI (kg/m^2^)	23.34 ± 2.25	22.50 ± 2.12	0.000
Hypertension, *n* (%)	98 (24.87%)	12 (9.23%)	0.000
Diabetes mellitus, *n* (%)	67 (17.01%)	10 (7.69%)	0.009
Atrial fibrillation, *n* (%)	62 (15.74%)	6 (4.62%)	0.001
Total cholesterol (mmol/L)	4.42 ± 0.95	4.28 ± 0.81	0.132
Triglyceride (mmol/L)	1.82 ± 1.33	1.86 ± 1.61	0.758
LDL-C (mmol/L)	3.09 ± 0.97	2.53 ± 0.74	0.000
HDL-C (mmol/L)	0.96 ± 0.26	0.99 ± 0.27	0.240
Creatinine (μmol/L)	78.92 ± 33.72	73.92 ± 30.19	0.133
FGF23 (pg/ml)	525.14 ± 167.40	338.62 ± 161.71	0.000
NIHSS	14.47 ± 8.20	—	—
mRS	2.45 ± 1.25	—	—
Stroke syndrome, *n* (%)			
TACS	91 (23.10%)	—	—
PACS	123 (31.22%)	—	—
LACS	70 (17.77%)	—	—
POCS	110 (27.92%)	—	—
Stroke etiology, *n* (%)			
Large-vessel occlusive	71 (18.02%)	—	—
Small-vessel occlusive	88 (22.34%)	—	—
Cardioembolic	89 (22.59%)	—	—
Multiple causes	84 (21.32%)	—	—
Unknown	62 (15.74%)	—	—

AIS, acute ischemic stroke; BMI, body mass index; LDL-C, low density lipoprotein cholesterol; HDL-C, high density lipoprotein cholesterol; FGF23, fibroblast growth factor 23; NIHSS, national institute of health stroke scale; mRs, modified rankin scale; TACS, total anterior circulation syndrome; PACS, partial anterior circulation syndrome; LACS, lacunar syndrome; POCS, posterior circulation syndrome.

### 3.2 Comparison of clinical data between the MACCE and non-MACCE groups of AIS patients

**Figure 1:**
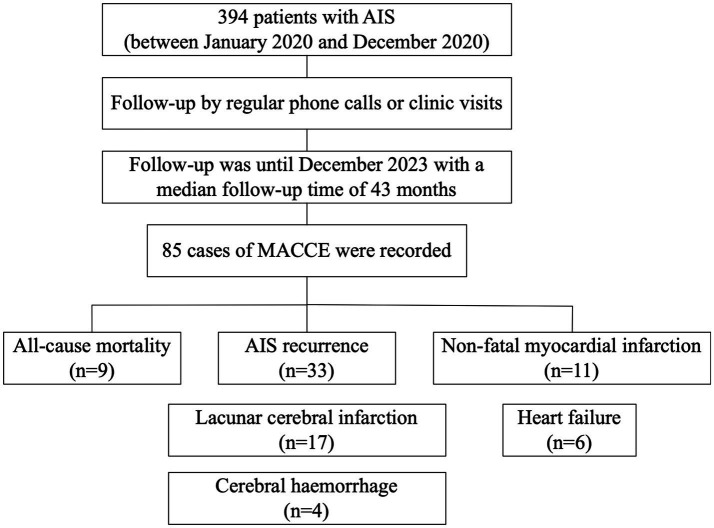
Flow chart of the study.

In this study, 394 patients with AIS were followed up for a median duration of 43 months (interquartile range: 39–44 months). By the end of follow-up, a total of 85 MACCEs were recorded, including 33 AIS recurrences, 17 lacunar cerebral infarctions, 4 cerebral haemorrhages, 11 non-fatal myocardial infarctions, 6 heart failures and 9 all-cause deaths (Figure 1). Patients with AIS were divided into a MACCE group and a non-MACCE group based on whether a MACCE occurred. A comparison of the demographic and baseline clinical characteristics between the two groups is shown in Table 2.

**Table 2:** The Comparison of clinical data between the MACCE and no MACCE group in AIS patients.

Variables	MACCE group (*n* = 85)	No MACCE group (*n* = 309)	*p*
Male, *n* (%)	53 (62.35%)	183 (59.22%)	0.602
Age (years)	70.95 ± 12.93	66.32 ± 11.94	0.002
BMI (kg/m^2^)	23.67 ± 2.43	23.25 ± 2.19	0.125
Hypertension, *n* (%)	35 (41.18%)	63 (20.39%)	0.000
Diabetes mellitus, *n* (%)	19 (22.35%)	48 (15.53%)	0.138
Atrial fibrillation, *n* (%)	29 (34.12%)	33 (10.68%)	0.000
Total cholesterol (mmol/L)	4.42 ± 0.90	4.42 ± 0.96	0.945
Triglyceride (mmol/L)	1.78 ± 1.08	1.83 ± 1.39	0.776
LDL-C (mmol/L)	3.48 ± 0.93	2.98 ± 0.96	0.000
HDL-C (mmol/L)	0.90 ± 0.22	0.98 ± 0.26	0.013
Creatinin (μmol/L)	80.09 ± 35.69	78.60 ± 33.21	0.719
FGF23 (pg/ml)	646.09 ± 164.05	491.87 ± 152.54	0.000
NIHSS	21.26 ± 9.14	12.60 ± 6.84	0.000
mRS	3.69 ± 1.39	2.11 ± 0.96	0.000
Stroke syndrome, *n* (%)			
TACS	34 (40.00%)	57 (18.45%)	0.000
PACS	20 (23.53%)	103 (33.33%)	0.084
LACS	12 (14.12%)	58 (18.77%)	0.320
POCS	19 (22.35%)	91 (29.45%)	0.196
Stroke etiology, *n* (%)			
Large-vessel occlusive	12 (14.12%)	59 (19.09%)	0.290
Small-vessel occlusive	19 (22.35%)	69 (22.33%)	0.996
Cardioembolic	28 (32.94%)	61 (19.74%)	0.010
Multiple causes	16 (18.82%)	68 (22.01%)	0.526
Unknown	10 (11.76%)	52 (16.83%)	0.256

MACCE, major adverse cardiovascular and cerebrovascular events; AIS, acute ischemic stroke; BMI, body mass index; LDL-C, low density lipoprotein cholesterol; HDL-C, high density lipoprotein cholesterol; FGF23, fibroblast growth factor 23; NIHSS, national institute of health stroke scale; mRs, modified rankin scale; TACS, total anterior circulation syndrome; PACS, partial anterior circulation syndrome; LACS, lacunar syndrome; POCS, posterior circulation syndrome.

Serum FGF23 levels were significantly greater in the MACCE group than in the non-MACCE group (646.09 ± 164.05 pg./mL vs. 491.87 ± 152.54 pg./mL, *p* < 0.05). Compared with the non-MACCE group, the MACCE group presented higher NIHSS and mRS scores (*p* < 0.05). The prevalence of total anterior circulation syndrome and cardioembolic events was markedly greater in the MACCE group than in the non-MACCE group (*p* < 0.05). There was a statistical difference between the two groups in terms of age, history of hypertension, history of atrial fibrillation, and blood LDL cholesterol and HDL cholesterol levels, whereas there was no statistically significant difference in terms of sex, BMI, history of diabetes mellitus, or blood levels of total cholesterol, triglycerides and creatinine.

### 3.3 ROC analyses to predict the occurrence of MACCEs in AIS patients

**Figure 2:**
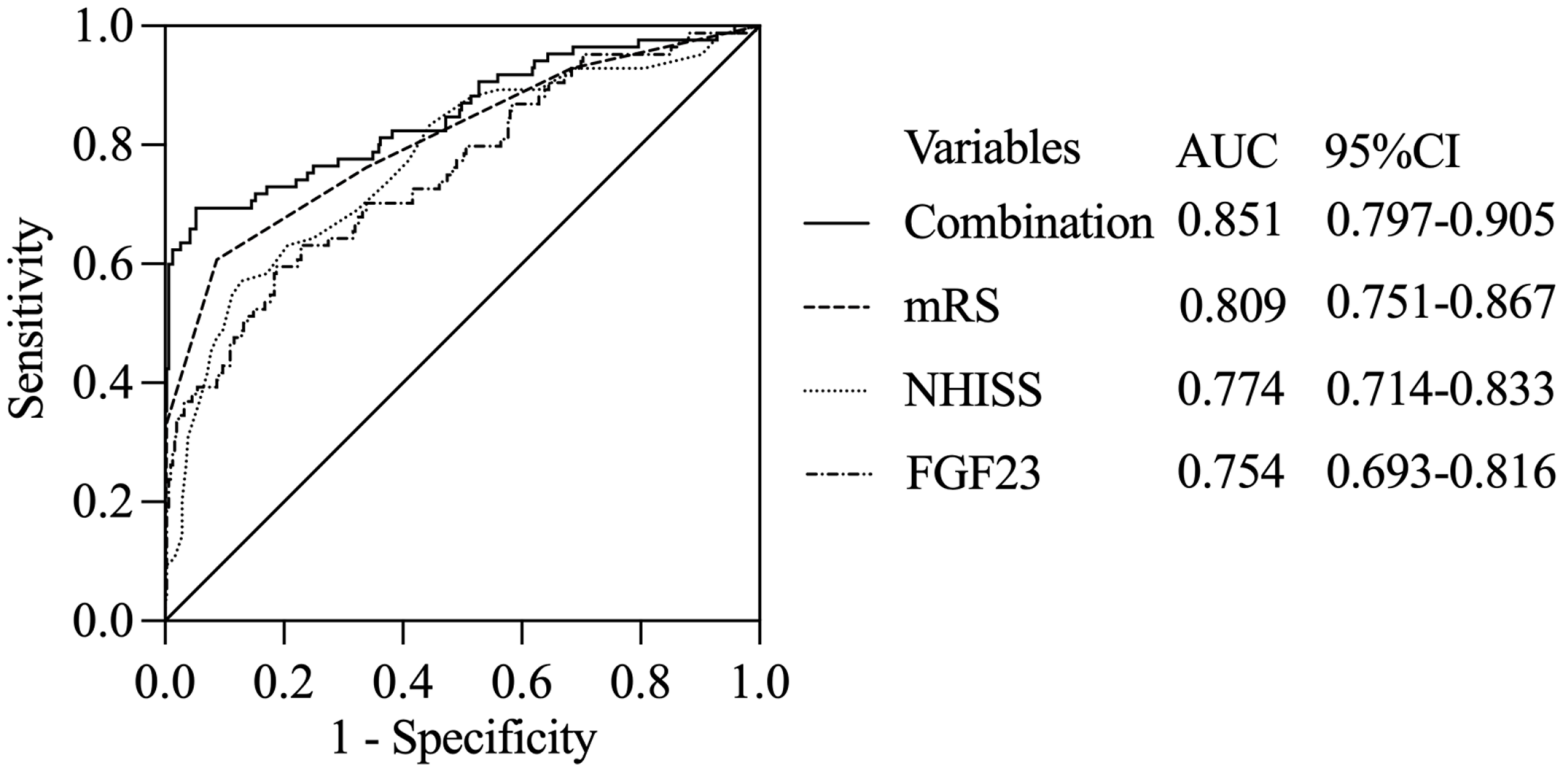
ROC analyses to predict the occurrence of MACCE in AIS patients.

To assess the value of FGF23 in predicting the occurrence of MACCE in patients with AIS, an ROC analysis was performed, and the NIHSS score and mRS score were selected for comparison (Figure 2). According to the ROC analysis, the area under the curve (AUC) of FGF23 was 0.754 (95% CI: 0.693–0.816), which was lower than those of the NIHSS score (AUC: 0.774, 95% CI: 0.714–0.833) and mRS score (AUC: 0.809, 95% CI: 0.751–0.867). However, the difference among the three factors was not statistically significant. The AUC of the combination of the three factors was 0.851 (95% CI: 0.797–0.905), which was greater than that of the mRS score, NIHSS score, and FGF23 score (Z: 2.557, 3.051, and 3.679, respectively; all *p* < 0.05).

### 3.4 Kaplan–Meier analysis of FGF23 and the occurrence of MACCEs in AIS patients

**Figure 3:**
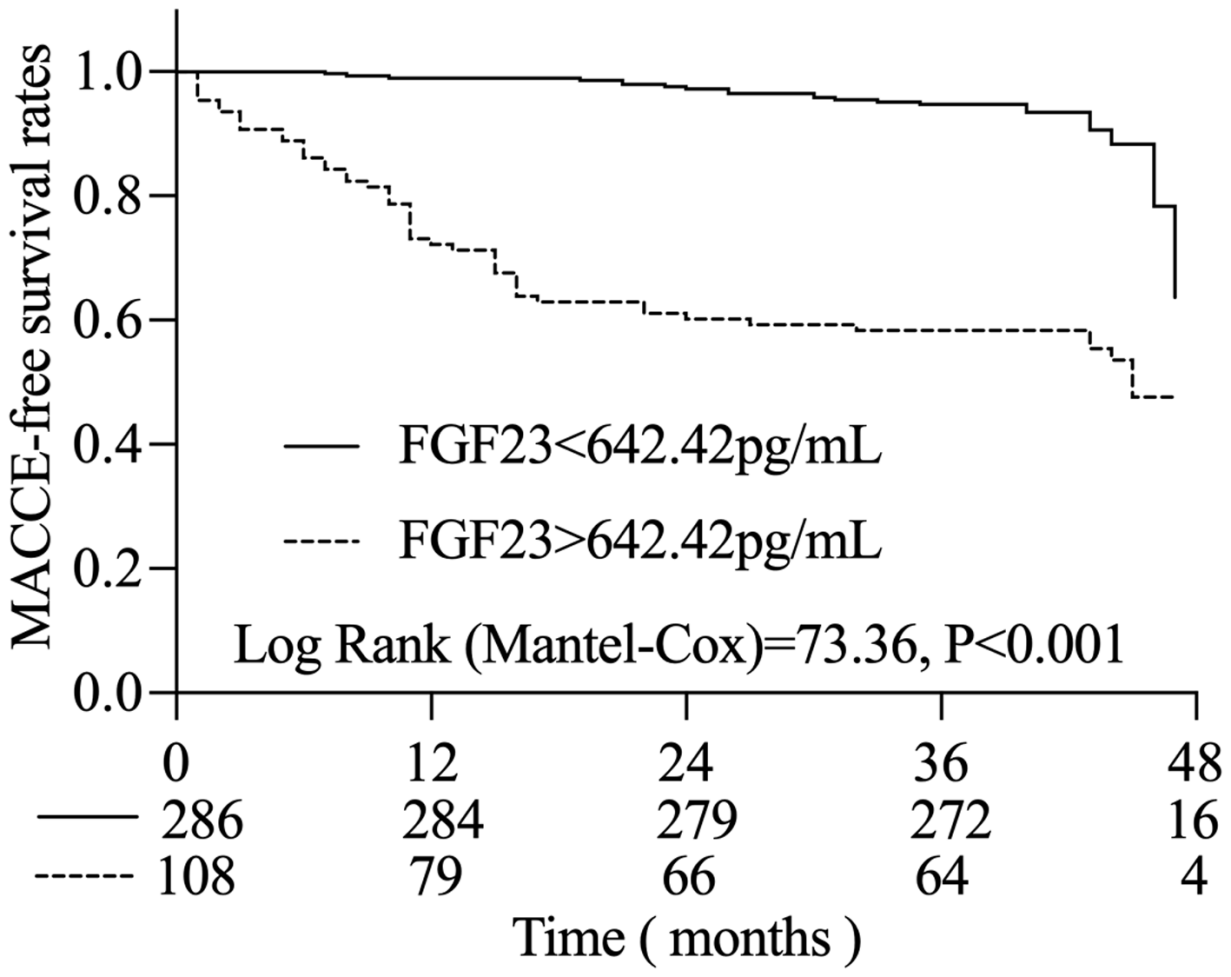
Kaplan–Meier analysis of FGF23 and the occurrence of MACCE in AIS patients.

The optimal cut-off value of FGF23 (642.42 pg./mL) based on the Jordon’s index was then used to perform survival analyses. A Kaplan–Meier analysis based on this cut-off value revealed a significant difference between the two groups in terms of cumulative survival rates without MACCEs (Figure 3). The cumulative MACCE-free survival rate was significantly greater in the low FGF23 level group than in the high FGF23 level group (*p* < 0.05). Moreover, the difference between the two groups gradually increased over time, indicating that high FGF23 levels were associated with lower MACCE-free survival.

### 3.5 Univariate and multivariate COX analysis for MACCEs in AIS patients

The univariate COX analysis revealed that elevated baseline serum FGF23 levels with advanced age, elevated BMI, a previous history of hypertension and atrial fibrillation, elevated LDL-C levels, high NIHSS and mRS scores, and stroke syndrome were predictive factors for the development of MACCEs in AIS patients (Table 3). Further multivariate Cox analyses of statistically significant metrics revealed that elevated baseline serum FGF23 levels (HR: 3.731, 95% CI:2.157–6.452) remained a potent predictor of the occurrence of MACCEs in patients with AIS, as did advanced age, a history of previous AF, elevated LDL-C levels, high NIHSS and mRS scores, and stroke syndrome.

**Table 3:** Univariate and multivariate COX analysis for MACCE in AIS patients.

Variables	Univariate analysis			Multivariate analysis		
	HR	95% CI	*p*	HR	95% CI	*p*
Sex (male)	1.204	0.775–1.868	0.409	—	—	—
Age (years)	1.668	1.084–2.567	0.020	1.795	1.099–2.931	0.019
BMI	1.718	1.083–2.727	0.022	—	—	—
Hypertension	2.119	1.375–3.265	0.001	—	—	—
Diabetes mellitus	1.457	0.875–2.428	0.148	—	—	—
Atrial fibrillation	3.036	1.937–4.757	0.000	2.342	1.353–4.053	0.002
Total cholesterol	1.772	0.855–3.671	0.124	—	—	—
Triglyceride	1.350	0.87–2.076	0.172	—	—	—
LDL-C	2.596	1.675–34.022	0.000	2.148	1.338–3.446	0.002
HDL-C	0.305	0.042–2.204	0.239	—	—	—
Creatinine	1.471	0.956–2.262	0.079	—	—	—
FGF23	5.551	3.591–8.582	0.000	3.731	2.157–6.452	0.000
NIHSS	8.013	5.140–12.493	0.000	4.287	2.521–7.290	0.000
mRS	10.614	6.834–16.485	0.000	5.388	3.061–9.484	0.000
Stroke syndrome (TACS vs. other)	2.999	1.940–4.635	0.000	3.120	1.869–5.209	0.000
Stroke etiology (Cardioembolic vs. other)	1.902	1.208–2.993	0.005	—	—	—

MACCE, major adverse cardiovascular and cerebrovascular events; AIS, acute ischemic stroke; BMI, body mass index; LDL-C, low density lipoprotein cholesterol; HDL-C, high density lipoprotein cholesterol; FGF23, fibroblast growth factor 23; NIHSS, national institute of health stroke scale; mRs, modified rankin scale; TACS, total anterior circulation syndrome.

## 4 Discussion

In the present study, serum FGF23 levels were found to be elevated in AIS patients. After a median follow-up of 43 months, serum FGF23 levels were further elevated in AIS patients who experienced MACCEs. FGF23 had similar high predictive value as the NIHSS score and mRS score for the occurrence of MACCEs in AIS patients. Moreover, elevated baseline serum FGF23 levels were an effective predictor of the occurrence of MACCEs in AIS patients.

Multiple factors involved in the development of AIS, such as the inflammatory response, hypoxia, energy and metabolism, promote FGF23 production (33, 34). FGF23, which is secreted by osteoblasts and osteoclasts, acts via endocrine forms in several organs throughout the body, including the kidneys, parathyroid glands, heart, bones and brain (14, 35). FGF23 not only inhibits the expression of NPT2a and NPT2c sodium-phosphate cotransporters, but also reduces 1,25(OH)2D levels to inhibit renal proximal tubular and intestinal phosphate reabsorption and thereby reduce serum phosphate levels (36).

As a key molecule in cellular metabolic processes, phosphate plays an important role in the development of AIS by participating in energy metabolism, cellular signal transduction, the inflammatory response, oxidative stress, and calcium overload (37–39). Reduced serum phosphate levels were strongly associated with the risk of developing AIS. A follow-up of 3,437 patients undergoing haemodialysis for a median of 3.9 years revealed that the lower the serum phosphate level was, the greater the risk of developing AIS (40). For AIS patients, reduced serum phosphate levels are associated not only with severity but also with the occurrence of MACCEs (41–43). The inhibition of FGF23 overactivity in certain diseases caused by hypophosphatemia is considered a new therapy for the treatment of these diseases, as the human monoclonal antibody (burosumab) for FGF23 has been approved in several countries including Europe, North America and Japan (36).

Elevated FGF23 was associated with vascular calcification, vascular stiffness and endothelial dysfunction, all of which may contribute to AIS (44). The FGF23 receptor Klotho, which is widely expressed in the brain, has anti-inflammatory and antioxidant effects, and its levels decrease with age and cardiovascular disease (45). Statins and renin-angiotensin system inhibitors, which are widely used in clinical practice, can increase Klotho levels and improve the prognosis of cardiovascular diseases (35).

Elevated serum FGF23 levels are associated with an increased risk of developing AIS in individuals without chronic kidney disease (21). Furthermore, in these individuals, FGF23 is not only independently associated with insulin resistance, inflammation, and obesity status but is also a strong predictor of MACCEs in patients with cardiovascular disease (46–48). In addition, many studies have shown that the NIHSS score and mRS score are predictors of the occurrence of MACCEs in AIS patients (32, 49). This finding is similar to our findings that FGF23 has the same value as the NIHSS score and mRS for predicting the occurrence of MACCEs in patients with AIS.

However, the MESA and REGARDS studies revealed that serum FGF23 levels were not associated with the risk of developing AIS or were only associated with only the risk of developing cardioembolic stroke (28, 29). The apparent discrepancies between our findings and those of previous population-based cohorts such as MESA and REGARDS may stem from critical differences in both study design and outcome definitions. While those studies assessed FGF23 as a risk factor for incident stroke in ostensibly healthy individuals, our work evaluates its prognostic value for recurrent or progressive cardiovascular and cerebrovascular events (i.e., MACCEs) in a well-characterized cohort of patients with established acute ischemic stroke. These distinct clinical contexts—primary prevention versus secondary event prediction—likely account for divergent associations. Moreover, methodological heterogeneity, including the use of different FGF23 immunoassays, variable adjustment for confounders, and diverse statistical approaches, further complicates direct comparisons across studies. Notably, FGF23 levels correlated with prevalent hypertension in our cohort (Supplementary Table S1), supporting the notion that its prognostic value may be context-dependent and amplified in secondary prevention settings. Future harmonized prospective investigations employing standardized assays and consistent outcome adjudication are needed to resolve these inconsistencies.

## 5 Conclusion

In conclusion, the present study demonstrated that serum FGF23 levels were elevated in patients with AIS. Baseline serum FGF23 levels exhibited high predictive value for MACCEs in AIS patients. Furthermore, elevated baseline serum FGF23 levels were identified as a significant and independent predictor of MACCE occurrence in AIS patients.

### 5.1 Limitations

There were several limitations to this study. First, this study was a single-centre study with patient enrolment in only one hospital in one region, which inevitably resulted in selection bias. Second, in this study, only the baseline serum FGF23 levels of the patients were tested, and the levels were not retested at the time of MACCE occurrence, which did not allow for a pre–post controlled study. Therefore, multicentre, large-scale, long-term clinical studies are still needed to explore the relationship between FGF23 levels and the risk of developing AIS. It is important to note that the study did not measure serum phosphate levels, a central component of the FGF23–Klotho–phosphate axis. This limitation precludes definitive conclusions about whether the prognostic value of FGF23 is mediated through phosphate dysregulation or represents a direct pathological effect.

## Data Availability

The raw data supporting the conclusions of this article will be made available by the authors, without undue reservation.
