# Supplementary material for: Elevated fibroblast growth factor 23 levels predict cardiovascular and cerebrovascular events in acute ischaemic stroke patients
Source: Front Neurol. 2026 Mar 20;17:1777221. doi: 10.3389/fneur.2026.1777221 (PMC13046549; doi:10.3389/fneur.2026.1777221)
Supplement: Supplementary file 1 [file Table_1.docx]

**TABLE S1** Serum FGF23 Levels in AIS Patients Stratified by Comorbidities

| Comorbidity | Subgroup | n | FGF23 (pg/ml) | P |
| --- | --- | --- | --- | --- |
| Hypertension | Present | 98 | 573.48±155.56 | 0.001 |
|  | Absent | 296 | 509.14±168.35 |  |
| Diabetes mellitus | Present | 67 | 555.22±168.31 | 0.107 |
|  | Absent | 327 | 518.98±166.80 |  |
| Atrial fibrillation | Present | 62 | 558.74±180.97 | 0.085 |
|  | Absent | 332 | 518.87±164.27 |  |

FGF23, fibroblast growth factor 23; AIS, acute ischemic stroke; BMI, body mass index.
